# Waterborne aripiprazole blunts the stress response in zebrafish

**DOI:** 10.1038/srep37612

**Published:** 2016-11-22

**Authors:** Heloísa Helena de Alcantara Barcellos, Fabiana Kalichak, João Gabriel Santos da Rosa, Thiago Acosta Oliveira, Gessi Koakoski, Renan Idalencio, Murilo Sander de Abreu, Ana Cristina Varrone Giacomini, Michele Fagundes, Cristiane Variani, Mainara Rossini, Angelo L Piato, Leonardo José Gil Barcellos

**Affiliations:** 1 Programa de Pós-Graduação em Farmacologia, Universidade Federal de Santa Maria (UFSM), Av. Roraima, 1000, Cidade Universitária, Camobi, Santa Maria, RS, 97105-900, Brazil; 2 Universidade de Passo Fundo (UPF), BR 285, São José, Passo Fundo, RS, 99052-900, Brazil; 3 Programa de Pós-Graduação em Farmacologia e Terapêutica, Instituto de Ciências Básicas da Saúde, Universidade Federal do Rio Grande do Sul, Porto Alegre, RS, 90050-170, Brasil; 4 Programa de Pós-Graduação em Bioexperimentação, Universidade de Passo Fundo (UPF), BR 285, São José, Passo Fundo, RS, 99052-900, Brazil

## Abstract

Here we provide, at least to our knowledge, the first evidence that aripiprazole (APPZ) in the water blunts the stress response of exposed fish in a concentration ten times lower than the concentration detected in the environment. Although the mechanism of APPZ in the neuroendocrine axis is not yet determined, our results highlight that the presence of APPZ residues in the environment may interfere with the stress responses in fish. Since an adequate stress response is crucial to restore fish homeostasis after stressors, fish with impaired stress response may have trouble to cope with natural and/or imposed stressors with consequences to their welfare and survival.

The consumption of antipsychotic drugs has been growing gradually, especially due to the increasing the number of diagnostics of psychotic disorders in recent decades^1^. These drugs induce serious extrapyramidal effects in patients who are treated in a continuous and prolonged manner^2^. In 2002, the US FDA approved the use of aripiprazole (APPZ), an atypical antipsychotic with considerably lower occurrence and intensity of adverse effects^3^,^4^. APPZ has been approved for the treatment of schizophrenia, bipolar mania and major depressive disorder^5^,^6^,^7^,^8^,^9^, and is useful for controling positive and negative symptoms in schizophrenia^10^, and irritability, hyperactivity and stereotypies in autism^11^.

Due to its safety and lower incidence of unwanted side effects, the prescription of APPZ is increasing to replace classical antipsychotics. This increased use justify the concern about the accumulation of APPZ residues in wastewater and, consequently, potential negative effects in non-target organisms such as fish. There are a few studies reporting APPZ concentrations detected in effluents^12^,^13^, and only one study of cardiovascular risk using zebrafish larvae^14^. In addition, there are no reports on the impact of this drug in adult zebrafish (*Danio rerio*).

Endocrine disruptors are compounds that alter the normal functioning of the endocrine system of both humans and wildlife. We have previously shown that other psychotropic drugs such as risperidone^15^, fluoxetine, diazepam^16^,^17^ as well as alcohol^18^ blunted the response to stress follow acute stress”.

Despite the knowledge on the effects of APPZ *in vitro*^18^,^19^,^20^, in rodents^21^,^22^ and humans^11^,^23^,^24^, little is known about its impact as a possible environmental contaminant. Coupled with the increasing use, interest in the study of psychiatry drugs as environmental contaminants^13^,^25^,^26^ and the impact they have on the aquatic fauna has risk^27^,^28^,^29^,^30^,^31^. Thus, here we describe, for the first time, the effects of different concentrations of APPZ on the hypothalamic-pituitary-interrenal (HPI) axis in adult zebrafish.

## Results

**Figure 1:**
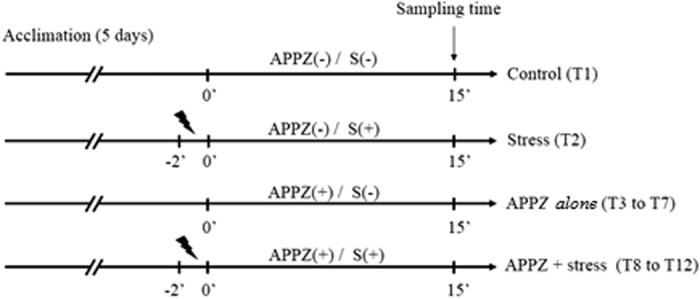
Whole-body cortisol levels in zebrafish exposed to different concentrations of aripiprazole (APPZ, in ng/L) without stressor stimulus (S−) and with stressor stimulus (S+). Each dot represents one independent measurement and the black lines represent the mean. Two-way ANOVA followed by Tukey post hoc test. n = 9–15 **p < 0.01; ***p < 0.001; ****p < 0.0001 vs. control group (S−).

Two-way ANOVA revealed significant main effects for stress (F_1,123_ = 10.33, p < 0.0001), as well as a significant interaction between the factors (F_5,123_ = 101.9, p < 0.0001), but not for drug (F_5,123_ = 1.54; p = 0.1822) (Fig. 1). At concentrations of 0.556 and 556 ng/L, APPZ prevented the increase in cortisol levels in stressed zebrafish. However, this effect was not observed at other concentrations. On the other hand, APPZ *per se* did not interfere with whole-body cortisol levels. We can also observe that the stressor stimulus was effective.

## Discussion

Here we provide, at least to our knowledge, the first evidence that APPZ in the water decreases the stress response of exposed fish. The concentration of 0.556 ng/L, ten times lower than the concentration detected in the environment (5.56 ng/L^*13*^), blunted the cortisol increase after an acute stressor. This blunting effect of the lower concentration, in an environmental perspective, points to the necessity of great caution in relation to the delivery of APPZ residues in water bodies.

Interestingly, the HPI-blunting effect was also evident in the higher concentrations of 556 ng/L. This U-shaped concentration-response curve suggests a hormetic effect, similar to other psychotropic drugs such as diazepam^16^ and risperidone^17^.

In fact, the mechanism of action of APPZ on the HPI axis of zebrafish is not clear. Our hypothesis is that APPZ has a dopaminergic stabilizer effect^20^, since it is a partial agonist of dopamine receptors^30^, and tends to buffer the effects of endogenous dopamine^31^. Since dopamine receptors regulate hypothalamic-pituitary-adrenal axis activity in rats^32^, it is possible that APPZ may also act via this mechanism in fish. Our hypothesis based on the dopaminergic regulation of HPI axis is reinforced since in unstressed fish, the APPZ did not cause any change in cortisol levels, as verified in humans^25^. In addition, zebrafish treated with a dopaminergic antagonist and subjected to stress did not increase their cortisol, reinforcing the dopaminergic involvement in the HPI activation^33^.

Despite the undetermined mechanism, our results highlight that the presence of APPZ residues in the environment may interfere in the neuroendocrine axis that coordinates the stress responses in fish. In fish, cortisol plays a key role in the intermediary metabolism, osmoregulation and immune function^34^. Thus, the adequate stress response is crucial to restoring fish homeostasis after stressors; a fish with an impaired response may have trouble to cope with natural and/or imposed stressors with consequences to fish welfare and survival.

A limitation of our study is that we evaluated the effects of acute, but not chronic exposition to AAPZ. Further studies are necessary to better understand the effects of chronic exposition of AAPZ on behavioral and neuroendocrine parameters in zebrafish.

Furthermore, the zebrafish is a model for translational studies of several human diseases, due to the high homology with the human genome^35^. Although we do not have addressed the mechanism whereby APPZ blocks the stress response, this does not lessen the importance of our initial assessment, since, at least to our knowledge, this is the first report about an *in vivo* APPZ effect in fish HPI axis.

## Methods

### Ethical note

This study was approved at protocol #20/2016, by the Ethics Commission for Animal Use of Universidade de Passo Fundo (Passo Fundo, RS, Brazil) and all methods were carried out in accordance with the guidelines of National Council of Animal Experimentation Control (CONCEA).

### Fish

We used a population of 144 mixed-sex adult wild-type zebrafish (*Danio rerio*), short-fin strain, and 50:50 male and female. Fish were acclimatized for 5 days in 3.9 l glass aquaria (20 × 15 × 14 cm), in groups of three animals, under constant aeration, with a photoperiod of 14 h light: 10 h dark. Water temperature was maintained at 27.4 ± 1.3 °C; pH 6.7 ± 0.3; dissolved oxygen at 5.6 ± 0.5 mg/L; total hardness at 58.9 ± 12.4 mg/L CaCO_3_, alkalinity at 45 ± 10 mg/L CaCO_3_ and ionized ammonia was <0.011 mg/L.

### Study strategy

Our strategy was to expose zebrafish to five concentrations of APPZ and verify the stress response of these fish to an additional acute stressor. A possible isolated effect of APPZ was accessed by measuring cortisol in exposed unstressed fish.

### Experimental procedures

We distributed fish in 12 groups. Each group consist of four glass aquaria with three animals each one (n = 12 per treatment). We then exposed fish to five concentrations of APPZ (Aristab^®^, Aché, Brazil). We set two lower and two higher concentrations (at 10-fold basis) in relation to the APPZ concentration already detected in the environment (5.56 ng/L^*13*^). Thus, the five concentrations were 0.0556; 0.556; 5.556; 55.6 and 556 ng/L.

In the exposed and stressed groups, we administered APPZ directly in the water for a 15 min exposure time and then we applied the acute stress stimulus by chasing fish with a pen net for 2 minutes. After 15 min the animals were euthanized for cortisol measurement (see Fig. 1).

After this period, the fish were gently captured and immediately euthanized with cold water, followed by decapitation and freeze of the whole body at liquid nitrogen for 30 s. Then, we stored samples at −20 °C for cortisol extraction, following the method described in Oliveira *et al*.^18^. The tissue extract was suspended in PBS, and whole-body cortisol was measured using a commercially available enzyme-linked immune sorbent (EIAgen CORTISOL test, Bio Chem Immuno Systems). This kit is fully validated for zebrafish extracts using the methodology described by Sink *et al*.^36^.

### Statistics

The data is expressed as mean ± standard error of mean (S.E.M). The normal distribution of the data was confirmed by Kolmogorov-Smirnov and Levene tests, and results analyzed by two-way ANOVA followed by Tukey’s post hoc test. Two-way ANOVA was used to identify the main effects of stress and treatment, as well as their interactions. Differences were considered significant at p < 0.05”.

## Additional Information

**How to cite this article**: Barcellos, H. H. A. *et al*. Waterborne aripiprazole blunts the stress response in zebrafish. *Sci. Rep.* **6**, 37612; doi: 10.1038/srep37612 (2016).

**Publisher’s note:** Springer Nature remains neutral with regard to jurisdictional claims in published maps and institutional affiliations.

## Figures and Tables

**Figure 2:**
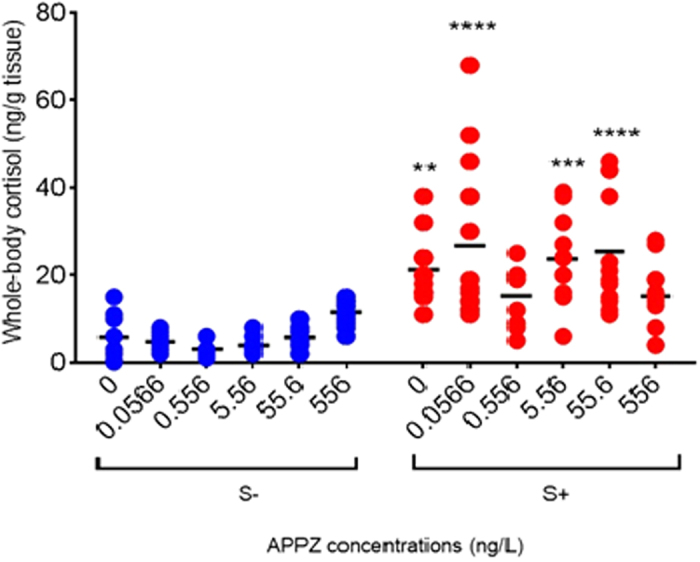
Schematic representation of the experimental design. APPZ (aripiprazole), S(−) without stressor stimulus; S(+) with stressor stimulus.
